# Supplementary material for: Multi-omics analysis of hospital-acquired diarrhoeal patients reveals biomarkers of enterococcal proliferation and Clostridioides difficile infection
Source: Nat Commun. 2023 Nov 25;14:7737. doi: 10.1038/s41467-023-43671-8 (PMC10676382; doi:10.1038/s41467-023-43671-8)
Supplement: Supplementary file 1 — Supplementary Information [file 41467_2023_43671_MOESM1_ESM.pdf]

Supplementary materials

Diarrhoeal specimen inclusion criteria

Inclusion criteria were as follows:

- Adequate (≥4 ml) excess stored sample after standard diagnostic testing was completed
- Only unformed samples (Bristol Stool Chart Category 5-6)
- First diarrhoea developed 48 hours after admission
- Adult patients > 18 years

Exclusion criteria were as follows:

- A specific pathogen (other than *C. difficile*) was identified from the diarrhoea faecal sample during routine diagnostic testing
- Formed stool
- Sample submitted was from outpatient or emergency departments
- Sample submitted < 48 hours after admission
- Repeat specimen (only first sample from patient's index admission processed)

FMT donor specimen inclusion criteria

For comparison, faecal samples were collected from healthy donors recruited for faecal microbiota transplantation (FMT) treatment of CDI. FMT is a unique treatment approach for recurrent CDI that aims to restore the commensal gut microbiota and in turn re-establish colonisation resistance to inhibit the growth of *C. difficile*. While high microbial diversity coupled with high proportions of SCFA and secondary bile acid producing bacteria are considered to be the key to successful FMT, little is known clinically about the role of amino acids and their fermentation products in *C. difficile* decolonisation. Most recently, studies have shown that FMT restoration of commensal microbiota increased competition for these preferred amino acids where species such as *Clostridium sardiniense*, with similar nutritional requirements as *C. difficile*, deplete amino acids in the gut to provide substantial protection against CDI [1]

The health status and eligibility of all donors were screened following BiomeBank's screening protocols that included an interview, medical assessment, blood, and stool screening. Donor samples in the study were obtained from 12 female and 8 male individuals, a smaller male cohort (40.0% male) compared to CDI patients (54.5% male) and non-CDI patients (47.4% male). FMT donors were between the ages of 18 and 46 with a

total age of 649 years and an average of 32.45 years. While the average age of FMT donors was significantly lower than the CDI (75 years, range 55-83) and non-CDI (68 years, range 52-78) median age, we chose FMT donors purposefully as a comparison group in order to assess the microbiota and metabolomes of CDI and non-CDI patients against FMT donors who are medically assessed as healthy and are actively recruited to treat recurrent and severe CDI.

- Recruited donors were assessed to have had no history of: Antimicrobial therapy or probiotics in the three months before donation
- Active medical illness or symptoms
- Medications (other than an oral contraceptive pill)
- International travel in the last six months
- High-risk sexual activity
- Illicit drug use
- Family history of colorectal carcinoma involving one or more first-degree relatives <55 years of age
- Household members with an active gastrointestinal infection

In addition, donors were medically assessed and had no record of:

- Any gastrointestinal disorder
- Obesity (BMI >30) and malnutrition (BMI <18)
- Hypertension
- Type 1 and 2 diabetes
- Autoimmune disease
- Depression
- Malignancy
- Stroke
- Heart disease

Stool screening conducted by BiomeBank included:

- Microscopy and culture
- Rotavirus, Norovirus and Adenovirus PCR
- *Clostridioides difficile* toxin PCR
- Egg, cysts, and parasites (including *Cryptosporidium* spp., *Giardia* spp., and *Entamoeba histolytica* PCR)
- Vancomycin-resistant *Enterococcus* (VRE) screen, Extended-spectrum beta-lactamase-producing Enterobacterales (ESBL), AmpC-producing Enterobacterales (AMP-C) and Carbapenem-resistant Enterobacteriaceae (CRE)
- Rectal swab testing for chlamydia, gonorrhoea, and herpes simplex virus DNA

Sample preparation, derivatisation and gas chromatography-mass spectrometry (GC-MS)

Freshly collected diarrhoeal samples were frozen at -80°C and freeze-dried. Metabolites were extracted and derivatised as previously described [2] with some modifications. Briefly, 40 mg of freeze-dried sample was homogenised with 990 µL of ice-cold methanol: water (6:4) mix. The samples were then centrifuged at 17,949 × g for 15 minutes at 4°C. The supernatant was transferred to fresh 2 mL centrifuge tubes. For pooled biological quality control (PBQC) mix, 10 µL aliquot was taken from each sample tube, and pooled in a new 10 mL Falcon tube. For the derivatisation purpose, 100 µL aliquots of faecal extract samples and PBQC samples were used. 1 µL of myristic acid-d27 (20.0 µg/mL in methanol) was added as an internal standard before each sample was vortexed for 2 minutes.

A quality control (QC) mix containing 19 different polar and semi-polar metabolites was prepared as per Fiehn (2016) [2-4]. The QC mix consisted of 500 ng dried metabolites of valine, succinic acid, methionine, 4-hydroxyproline, salicylic acid, α-ketoglutaric acid, shikimic acid, citric acid, lysine, glucose, sucrose, chlorogenic acid, myristic acid, myristic acid-d27, stigmasterol, glucose ($U^{13}C_6$), L-glutamine (Amide-^{15}N), palmitic acid (1-^{13}C) and glycine (1-^{13}C) (All standards were sourced from Novachem Pty. Ltd., Heidelberg West, VIC, Australia, unless specified otherwise) (Supplementary Table 8).

Prior to derivatisation faecal extract samples, PBQC mix, and QC mix were dried in a in a speed vacuum centrifuge (Model number: RVC 2-18; Martin Christ Gefriertrocknungsanlagen GmbH, Osterode, Germany) at 438 × g and 37°C. Derivatisation process was initiated by adding 10 µL methoxyamine HCl (20 mg/mL in pyridine) to each dried extract, followed by vortexing at 37°C in a ThermoMixer (ThermoMixer C, Eppendorf, Hamburg, Germany) at 1400 rpm for 90 minutes. Trimethylsilyl derivatisation was performed by adding 20 µL of N, O-Bis(trimethylsilyl) trifluoroacetamide (BSTFA) in 1% trimethylchlorosilane (TMCS) to the samples that were then incubated at 37°C in a ThermoMixer at 1400 rpm for 30 minutes [5]. Before GC-MS analysis, the derivatised samples were set aside at room temperature for 60 minutes.

Single quadrupole GC-MS analysis was performed, with retention time-locked to myristic acid-d27 internal standard, as described previously [6]. The analysis was performed using an Agilent 6890B GC oven coupled to a 5977B mass spectrometer detector (Agilent Technologies, Mulgrave, VIC, Australia) and fitted with a

multipurpose autosampler (Gerstel GmbH & Co.KG, Mulheim an der Ruhr, Germany). The GC-MS system was fitted with a 30 m HP-5MS column (0.25 mm internal diameter and 0.25 µm film thickness). 1 µL injections were performed in 1:10 split mode, with the oven held at an initial temperature of 70°C for 2 minutes. The temperature was then increased to 300°C at a rate of 7.5°C min−1 and held for 5 minutes. The transfer line was held at 280°C. Total ion chromatogram (TIC) mass spectra were acquired within a range of 50–650 m/z, with a 2.89 spectra s− 1 acquisition frequency. A solvent delay time of 6 minutes ensured that the source filament was not saturated or damaged. Total ion chromatogram (TIC) mass spectra were acquired within a range of 50–650 m/z, with a 2.89 spectra s-1 acquisition frequency. Data acquisition and spectral analysis for GC-MS were performed using the Qualitative Analysis software (Version B.010.00) of MassHunter workstation. Qualitative identification of the compounds was performed according to the Metabolomics Standard Initiative (MSI) chemical analysis workgroup using standard GC-MS reference metabolite libraries (Fiehn Metabolomics RTL Library, G166766A, Agilent Technologies).

Data was exported to Microsoft Excel®, where non-derivatised values and noise and background peak data were manually filtered out (See Supplementary Data). Peak values from extraction blanks were subtracted per batch run. Data were treated within the batch correction toolbox of MetaboAnalyst 4.0 to accommodate the differences in chromatograms across various timeframes within the same batch, and across various batches [7]. Missing values were replaced with 1/5 of the minimum observed area value for the given metabolite across each batch. Batch corrected data were normalised against the myristic acid-d27 internal standard (IS), with a relative standard deviation (RSD) of 12.3%. Normalisation was completed by multiplying the calculated error coefficient for myristic acid-d27 with the ratio of the compound peak area and the internal standard peak area for each sample. The resultant values were semi-quantitative (µg/µL).

$$\text{Error coefficient (IS)} = \text{Mass IS (µg)} \times \text{RSD IS (\%)}$$

$$\text{Normalised concentration (µg/µL)} = \text{Error coefficient (IS)} \times \frac{\text{Peak area of compound}}{\text{Peak area of IS}}$$

<u>SCFA sample preparation, derivatisation and Gas chromatography-mass spectrometry (GC-MS)</u>

Freshly collected diarrhoeal samples were frozen at -80°C and thawed before metabolite extraction. Metabolites were prepared and derivatized following the protocol developed by Furuhashi et al. [8], with some modifications. Briefly, 100-150 mg of thawed sample was homogenised in 1 mL of 10% isobutanol in sterile 1.5 mL homogenisation tubes (Navy RINO lysis kit, BioTools Pty. Ltd., Keperra, QLD, Australia) with zirconium oxide beads (0.15 mm and 0.5 mm) for 1 minute at 6500 rpm (Precellys Evolution Homogenizer; Bertin Technologies, Montigny-le-Bretonneux, France). Samples were then centrifuged at 16,000 x g at 4°C for 5 minutes. 675 µL of supernatant was transferred to a new tube, followed by 125 µL of 20 mM NaOH solution, 400 µL of chloroform and an internal standard of 5 µg/mL hexanoic acid-6,6,6-d3 (489727, Sigma Aldrich, USA) to monitor recoveries. The sample was vortexed and centrifuged at 16,000 x g at 4°C for 2 minutes. A 400 µL aliquot of the upper aqueous phase was transferred into a new tube, followed by 80 µL of isobutanol, 100 µL of pyridine and ultrahigh quality water to make up a 650 µL total volume. Quality control samples were created by pooling 10 µL of each faecal extract. Calibration standards of acetate, propionate, butyrate, isobutyrate and isovalerate were created at the following concentrations: 100 µg/mL, 50 µg/mL, 25 µg/mL, 12.5 µg/mL, and 7.25 µg/mL. An acetate, propionate, butyrate, isobutyrate and isovalerate quality control standard mix of 50 µg/mL was also created. Pooled samples, calibration standards, quality control mixes, extraction and derivatisation blanks for quality control were also topped up with 80 µL isobutanol and 100 µL pyridine and ultrahigh quality water to adjust to a 650 µL total volume.

50 µL isobutyl chloroformate was added to 650 µL of each faecal sample, the pooled faecal sample, and the SCFA standard solution. The tube lid was kept open for 1 minute to release the gases generated by the reaction. The samples were vortexed, followed by the addition of 150 µL hexane, and centrifuged at 21,000 x g at 4°C for 2 minutes. The upper phase (100 µL) was subsequently transferred to clean GC autosampler vials fitted with salinised glass inserts. An aliquot of malathion (1 µL, equivalent to 2.5 µg dry weight) was added to each sample as an internal standard to monitor machine performance.

The GC-MS analysis was performed on an Agilent 6890B gas chromatograph (GC) oven coupled to a 5977B mass spectrometer (MS) detector (Agilent Technologies, Mulgrave, VIC, Australia) fitted with an multipurpose (MPS) autosampler (Gerstel GmbH and Co.KG, Mülheim an der Ruhr, Germany). The GC oven

was fitted with two 15 m HP-5MS columns (0.25 mm ID and 0.25 µm film thickness; 19091S-431 UI, Agilent Technologies, Mulgrave, VIC, Australia), coupled to each other through a purged ultimate union (PUU) for the use of post-run back-flushing. The sample (1.0 µL) was introduced via a multimode inlet (MMI) operated in split mode (1:20). The column was maintained at 40 °C for 5 min, followed by an increase to 250 °C at a rate of 10 °C/min. This was followed by a second increment to 310 °C at a rate of 60 °C/min. The column was held at 310 °C for 1 min. The mass spectrometer was kept in extractor ion mode (EI mode) at 70 eV. The GC-MS ion source temperature and transfer line were kept at 250 and 280 °C, respectively. Detector voltage was kept at 1054 V. The MS detector was turned off for the first 3 min and, at 4.0–4.8 min and 12.5–13.2-min time windows until the excess derivatization reagent and chloroformate/hexane solvents were eluted from the column. This ensured that the source filament was not saturated and damaged. The scan range was kept in the range of *m/z* 35–350 (35–350 Daltons). Data acquisition and spectral analysis were performed as described in our previous study (Ref 8) and qualitative identification of metabolites was performed according to the Metabolomics Standard Initiative (MSI) chemical analysis workgroup using standard GC-MS reference metabolite libraries (NIST 17, Agilent Fiehn RTL Library [G166766A, Agilent Technologies] with the use of Kovats retention indices based on a reference n-alkane standard (C8-C40 Alkanes Calibration Standard, Sigma-Aldrich, Castle Hill, NSW, Australia).

Data acquisition and spectral analysis for GC-MS were performed using the Qualitative Analysis software (Version B.010.00) of MassHunter workstation. Qualitative identification of the compounds was performed according to the Metabolomics Standard Initiative (MSI) chemical analysis workgroup using standard GC-MS reference metabolite libraries (Fiehn Metabolomics RTL Library, G166766A, Agilent Technologies). Calibration curves were generated for each SCFA in GraphPad Prism 8.2.1 using calculated area values for standards. Linear regression of faecal SCFA area values was performed in GraphPad Prism 8.2.1 to determine concentrations. Each sample was weight normalised against the original fresh weight sample with concentrations expressed in µg/mg. Weight normalised SCFA concentrations were assessed in GraphPad Prism version 8.2.1 for Windows using the Mann-Whitney U and Kruskal-Wallis H tests, FDR adjusted for multiple comparisons using the Benjamini and Hochberg method.

Supplementary Table 1. Antibiotic combinations administered to hospital-acquired diarrhoeal patients before specimen collection

Antibiotic classes	+AAD Patients (n=137)	Number of classes	% +AAD patients
Aminoglycoside	1	1	0.7
Aminoglycoside + Cephalosporin + Cyclic lipopeptide + Nitroimidazole + Penicillin	1	5	0.7
Aminoglycoside + Cephalosporin + Fluoroquinolone	1	3	0.7
Aminoglycoside + Cephalosporin + Macrolide + Penicillin	1	4	0.7
Aminoglycoside + Cephalosporin + Macrolide + Penicillin + b-lactamase inhibitor	1	4	0.7
Aminoglycoside + Cephalosporin + Nitroimidazole	2	3	1.5
Aminoglycoside + Cephalosporin + Penicillin + Penicillin with β-lactamase inhibitor	1	4	0.7
Aminoglycoside + Glycopeptide	1	2	0.7
Aminoglycoside + Macrolide + Penicillin + Penicillin with β-lactamase inhibitor	1	4	0.7
Aminoglycoside + Penicillin	1	2	0.7
Carbapenem	2	1	1.5
Carbapenem + Cephalosporin + Glycopeptide + Nitroimidazole	1	4	0.7
Carbapenem + Cephalosporin + Glycopeptide + Penicillin	1	4	0.7
Carbapenem + Cephalosporin + Macrolide	1	3	0.7
Carbapenem + Cephalosporin + Macrolide + Penicillin with β-lactamase inhibitor	1	4	0.7
Carbapenem + Fluoroquinolone + Glycopeptide	1	3	0.7
Carbapenem + Glycopeptide	2	2	1.5
Carbapenem + Glycopeptide + Macrolide + Penicillin with β-lactamase inhibitor	1	4	0.7
Carbapenem + Glycopeptide + Penicillin with β-lactamase inhibitor	1	3	0.7
Carbapenem + Penicillin with β-lactamase inhibitor	1	2	0.7
Cephalosporin	13	1	9.5
Cephalosporin + Cyclic lipopeptide + Glycopeptide + Oxazolidinone + Penicillin + Penicillin with β-lactamase inhibitor	1	6	0.7
Cephalosporin + Fluoroquinolone	1	2	0.7
Cephalosporin + Fluoroquinolone + Lincosamide	1	3	0.7
Cephalosporin + Fluoroquinolone + Macrolide	1	3	0.7
Cephalosporin + Fluoroquinolone + Nitroimidazole + Penicillin with β-lactamase inhibitor	1	4	0.7
Cephalosporin + Glycopeptide + Penicillin with β-lactamase inhibitor	2	3	1.5
Cephalosporin + Macrolide	4	2	2.9
Cephalosporin + Macrolide + Nitroimidazole + Penicillin + Penicillin with β-lactamase inhibitor + Tetracycline	1	6	0.7
Cephalosporin + Macrolide + Penicillin + Penicillin with β-lactamase inhibitor	1	4	0.7
Cephalosporin + Macrolide + Tetracycline	1	3	0.7
Cephalosporin + Macrolide + Nitroimidazole	1	3	0.7
Cephalosporin + Nitroimidazole	19	2	13.9
Cephalosporin + Nitroimidazole + Penicillin with β-lactamase inhibitor	7	3	5.1
Cephalosporin + Nitroimidazole + Pyrimidine inhibitor	1	3	0.7
Cephalosporin + Nitroimidazole + Tetracycline	1	3	0.7
Cephalosporin + Penicillin	2	2	1.5

Antibiotic classes	+AAD Patients (n=137)	Number of classes	% +AAD patients
**Cephalosporin + Penicillin with β-lactamase inhibitor**	3	2	2.2
**Cephalosporin + Penicillin with β-lactamase inhibitor + Nitroimidazole**	1	3	0.7
**Cephalosporin + Penicillin + Penicillin with β-lactamase inhibitor**	1	3	0.7
**Cephalosporin + Penicillin + Penicillin with β-lactamase inhibitor + Tetracycline**	1	4	0.7
**Cephalosporin + Tetracycline**	1	2	0.7
**Cyclic lipopeptide + Fluoroquinolone + Nitroimidazole**	1	3	0.7
**Fluoroquinolone**	1	1	0.7
**Fluoroquinolone + Lincosamide**	1	2	0.7
**Fluoroquinolone + Lincosamide + Penicillin with β-lactamase inhibitor**	1	3	0.7
**Fluoroquinolone + Penicillin**	2	2	1.5
**Glycopeptide + Nitroimidazole + Penicillin with β-lactamase inhibitor**	2	3	1.5
**Glycopeptide + Penicillin**	1	2	0.7
**Glycopeptide + Penicillin with β-lactamase inhibitor**	2	2	1.5
**Glycopeptide + Penicillin with β-lactamase inhibitor + RNA synthesis Inhibitor**	1	3	0.7
**Macrolide**	2	1	1.5
**Macrolide + Penicillin**	1	2	0.7
**Macrolide + Penicillin with β-lactamase inhibitor**	2	2	1.5
**Penicillin**	2	1	1.5
**Penicillin with β-lactamase inhibitor**	25	1	18.2
**Penicillin + Tetracycline**	2	2	1.5
**Pyrimidine inhibitor**	3	1	2.2

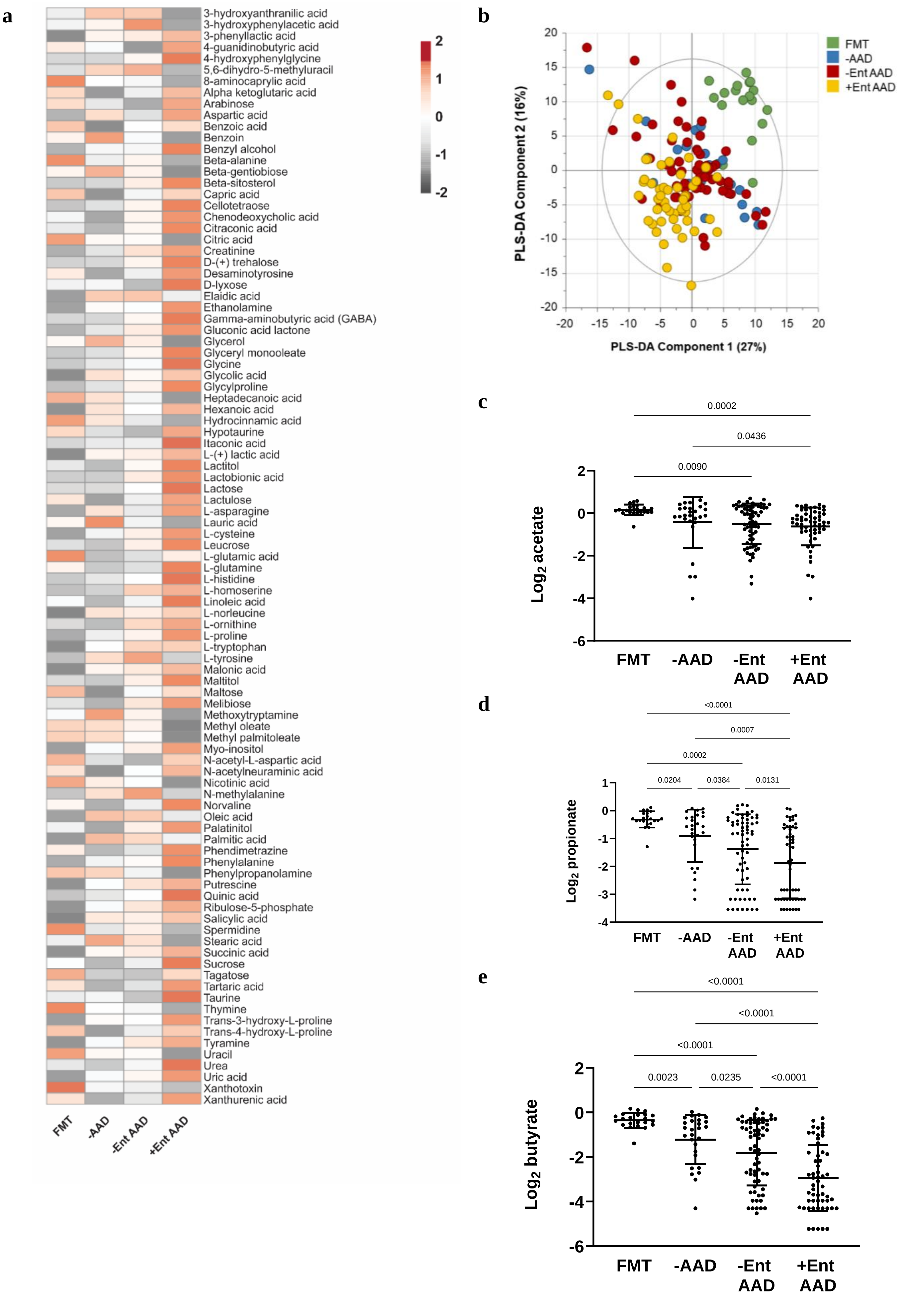
a
3-hydroxyanthranilic acid
3-hydroxyphenylacetic acid
3-phenyllactic acid
4-guanidinobutyric acid
4-hydroxyphenylglycine
5,6-dihydro-5-methyluracil
8-aminocaprylic acid
Alpha ketoglutaric acid
Arabinose
Aspartic acid
Benzoic acid
Benzoin
Benzyl alcohol
Beta-alanine
Beta-gentiobiose
Beta-sitosterol
Capric acid
Cellotetraose
Chenodeoxycholic acid
Citraconic acid
Citric acid
Creatinine
D-(+) trehalose
Desaminotyrosine
D-lyxose
Elaidic acid
Ethanolamine
Gamma-aminobutyric acid (GABA)
Gluconic acid lactone
Glycerol
Glyceryl monooleate
Glycine
Glycolic acid
Glycylproline
Heptadecanoic acid
Hexanoic acid
Hydrocinnamic acid
Hypotaurine
Itaconic acid
L-(+) lactic acid
Lactitol
Lactobionic acid
Lactose
Lactulose
L-asparagine
Lauric acid
L-cysteine
Leucrose
L-glutamic acid
L-glutamine
L-histidine
L-homoserine
Linoleic acid
L-norleucine
L-ornithine
L-proline
L-tryptophan
L-tyrosine
Malonic acid
Maltitol
Maltose
Melibiose
Methoxytryptamine
Methyl oleate
Methyl palmitoleate
Myo-inositol
N-acetyl-L-aspartic acid
N-acetylneuraminic acid
Nicotinic acid
N-methylalanine
Norvaline
Oleic acid
Palatinitol
Palmitic acid
Phendimetrazine
Phenylalanine
Phenylpropanolamine
Putrescine
Quinic acid
Ribulose-5-phosphate
Salicylic acid
Spermidine
Stearic acid
Succinic acid
Sucrose
Tagatose
Tartaric acid
Taurine
Thymine
Trans-3-hydroxy-L-proline
Trans-4-hydroxy-L-proline
Tyramine
Uracil
Urea
Uric acid
Xanthotoxin
Xanthurenic acid
FMT
-AAD
-Ent AAD
+Ent AAD
2
1
0
-1
-2
b
PLS-DA Component 2 (16%)
PLS-DA Component 1 (27%)
20
15
10
5
0
-5
-10
-15
-20
FMT
-AAD
-Ent AAD
+Ent AAD
c
0.0002
0.0436
0.0090
Log2 acetate
2
0
-2
-4
-6
FMT
-AAD
-Ent AAD
+Ent AAD
d
<0.0001
0.0007
0.0002
0.0204
0.0384
0.0131
Log2 propionate
1
0
-1
-2
-3
-4
FMT
-AAD
-Ent AAD
+Ent AAD
e
<0.0001
<0.0001
<0.0001
0.0023
0.0235
<0.0001
Log2 butyrate
2
0
-2
-4
-6
FMT
-AAD
-Ent AAD
+Ent AAD

Supplementary Figure 1. AAD with enterococcal proliferation was enriched in metabolites across several classes and depleted in short-chain and branched-chain fatty acids, forming a metabolomically distinct subset of AAD. a Heatmap of 97 PLS-DA derived metabolites differentiating FMT donors (n=18), -AAD (n=23), -Ent AAD (n=59) and +Ent AAD (n=51). All metabolites were normalised, Pareto scaled, and log-transformed. Each metabolite loading was scaled as a correlation co-efficient p(corr) between metabolomics models and the original data. Metabolites with VIP scores > 1.0 and p(corr) values > 0.5 and < -0.5 were identified as a subset of metabolites with the highest potential as biomarkers. For detailed VIP and p(corr) values, see Source Data file. **b** PLS-DA scores plot using 294 metabolites detected via untargeted GC-MS-based metabolomic profiling for FMT donors (green), -AAD (blue), -Ent AAD (red) and +Ent AAD (yellow) patients. Each point represented an individual specimen. PLS-DA model performance was cross-validated internally with R^2 and Q^2, CV-ANOVA, and multivariate ROC-AUC ($R^2Y=0.312$ and $Q^2=0.237$, $p=4.5 \times 10^{-5}$). For modelling details, see Source Data file. **c-e** Mean faecal GC-MS detected SCFA concentrations (acetate, propionate and butyrate) (µg per mg of fresh weight specimen (FW)) for FMT donors (n=20), -AAD (n=27), -Ent AAD (n=66) and +Ent AAD (n=56) patients. Data are presented as mean ± SD. Statistical significance was determined at $p< 0.05$ and comparisons used Kruskal-Wallis tests with FDR adjusted for multiple comparisons using the Benjamini and Hochberg method.

1

Supplementary Table 2. Top ROC-AUC derived metabolite biomarkers (AUC ≥ 0.7) that differentiated AAD with enterococcal proliferation from AAD without enterococcal proliferation.

Metabolite	AUC	P-value (t-test)
**Benzoin**	0.802	2.720×10^{-7}
**L-tyrosine**	0.791	2.965×10^{-7}
**3-hydroxyanthranilic acid**	0.777	7.291×10^{-7}
**Nicotinic acid**	0.734	3.444×10^{-5}
**Desaminotyrosine**	0.730	1.860×10^{-5}
**Chenodeoxycholic acid (CDCA)**	0.723	1.008×10^{-4}
**Benzyl alcohol**	0.706	9.755×10^{-5}
**Sucrose**	0.702	0.0012
**Uracil**	0.701	0.0003
**Citraconic acid**	0.701	0.0003

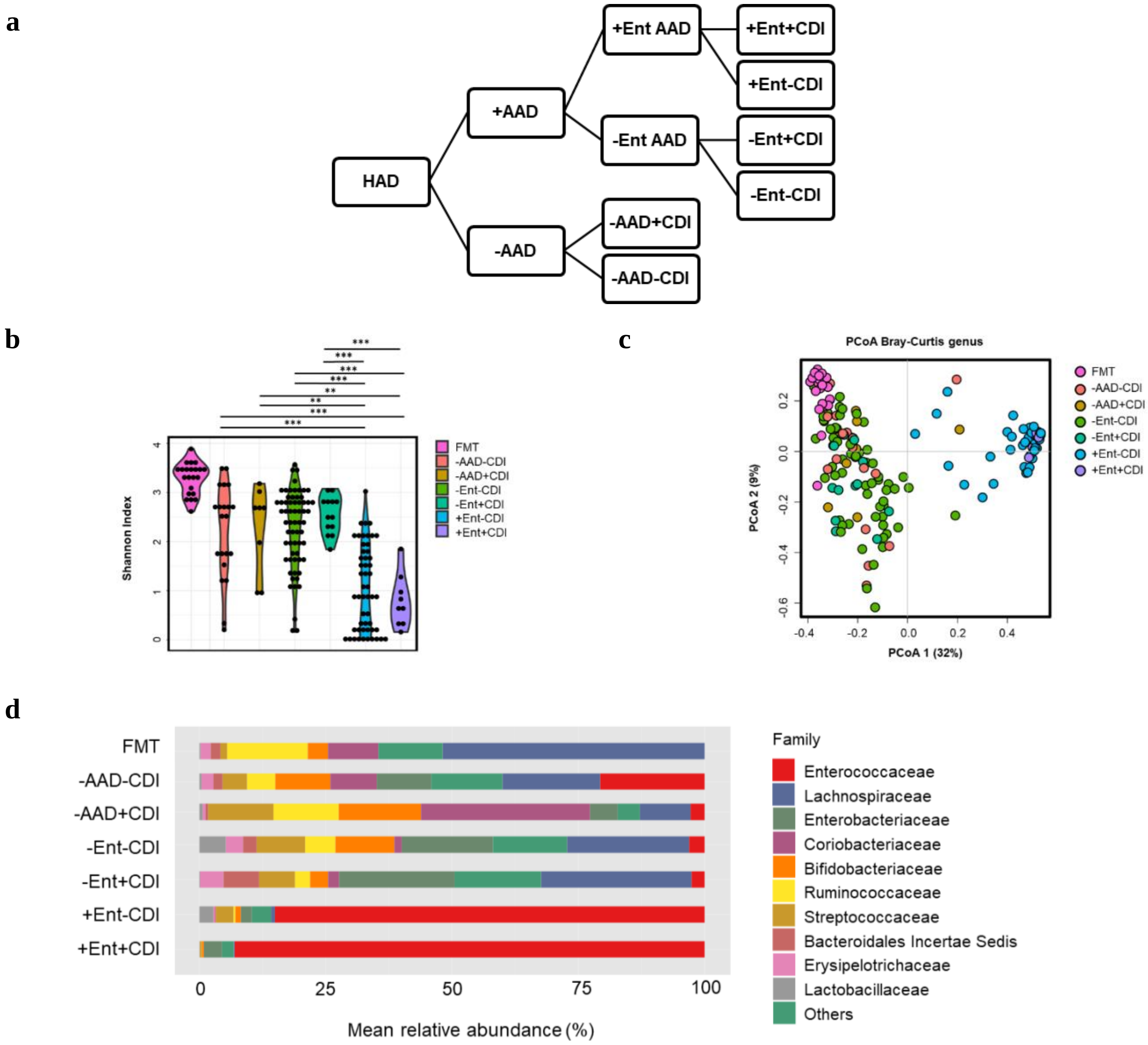

Supplementary Figure 2. CDI patients were associated with a spectrum of microbiota dysbiosis but were not microbially distinct from non-CDI patients. a Summary of patient cohorts in the study. Toxigenic *C. difficile* detected in 27% (9/33) of -AAD, 42% (14/33) of -Ent AAD and 30% (10/33) of +Ent AAD patients. Stratification resulted in the following groups: non-antibiotic associated non-CDI (-AAD-CDI), non-antibiotic associated CDI (-AAD+CDI), non-enterococcal dominant antibiotic-associated non-CDI (-Ent-CDI), non-enterococcal dominant antibiotic-associated CDI (-Ent+CDI), enterococcal-dominant antibiotic-associated non-CDI (+Ent-CDI) and enterococcal-dominant antibiotic-associated CDI (+Ent+CDI). **b** Violin plot of Shannon diversity indices that assessed species richness and evenness among FMT donors (pink), -AAD-CDI (orange), -AAD+CDI (olive), -Ent-CDI (green), -Ent+CDI (turquoise), +Ent-CDI (blue) and +Ent+CDI (purple) patients. Alpha diversity was estimated by the Shannon diversity index (rarefied to 1107 reads).

Statistical significance was determined at $p < 0.05$ and annotated by * $p < 0.05$, ** $p < 0.01$ and *** $p < 0.001$. Comparisons used Kruskal-Wallis tests with FDR adjusted for multiple comparisons using the Benjamini and Hochberg method. **c** PCoA plot based on Bray-Curtis dissimilarity assessed microbiota differences between FMT donors (pink), -AAD-CDI (orange), -AAD+CDI (olive), -Ent-CDI (green), -Ent+CDI (turquoise), +Ent-CDI (blue) and +Ent+CDI (purple) patients (R^2=0.303, $p < 0.001$). Statistical significance was determined at $p < 0.05$ by PERMANOVA. The F statistic two-tailed p-value depicts the significance of the host factor in affecting the community structure, while the PERMANOVA statistic R^2 depicts the fraction of variance explained by each factor. **d** Mean abundance of major taxa present in FMT donors, -AAD-CDI, -AAD+CDI, -Ent-CDI, -Ent+CDI, +Ent-CDI and +Ent+CDI patients, colour coded and presented as stacked bar graphs. Panels **a-d** represent FMT donors (n=20), -AAD-CDI (n=21), -AAD+CDI (n=8), -Ent-CDI (n=126), -Ent+CDI (n=13), +Ent-CDI (n=52) and +Ent+CDI (n=9).

Data in panel **b** is presented as mean ± SD. Statistical significance was determined at $p < 0.05$ and comparisons used Kruskal-Wallis tests with FDR adjusted for multiple comparisons using the Benjamini and Hochberg method.

CDI and non-CDI microbiota

Toxigenic *C. difficile* was detected in -AAD, -Ent AAD and +Ent AAD patients. CDI microbiota and metabolome analyses were grouped by antibiotic treatment and enterococcal dominance (Supplementary Figure 2A). There was no significant microbiota difference between CDI and non-CDI patients with respect to antibiotic treatment and enterococcal-dominance. Alpha diversity did not differ significantly between the CDI and non-CDI microbiota (Supplementary Figure 2B). All CDI and non-CDI groups showed a considerable spread and there was no significant difference in Shannon diversity between -AAD-CDI vs -AAD+CDI patients (p=0.346), -Ent-CDI vs -Ent+CDI patients (p=0.200) and +Ent-CDI vs +Ent+CDI patients (Supplementary Figure 2B). Furthermore, pairwise post-hoc PERMANOVA determined that there was no significant difference between CDI and non-CDI patients with a statistically insignificant distribution of centroids for -AAD-CDI vs -AAD+CDI patients (R^2=0.031, p=0.648), -Ent-CDI vs -Ent+CDI patients (R^2=0.015, p=0.302) and +Ent-CDI vs +Ent+CDI patients (R^2=0.019, p=0.248) (Supplementary Figure 2C).

Lastly, the mean abundance of major genera present among FMT donors, CDI and non-CDI patients revealed little difference between groups at a genus level (Supplementary Figure 2D).

Supplementary Table 3. Cross-validated CDI PLS-DA models with respect to antibiotic exposure and enterococcal proliferation

PLS-DA Classification Model	Number of model components	R2X	R2Y	Q2	P-value CV-ANOVA	Multivariate ROC-AUC
**CDI vs non-CDI**	3	0.472	0.489	0.144	Nil	AUC(-CDI)=0.773<br>AUC(+CDI)=0.944
**CDI vs non-CDI (stratified groups)**	4	0.519	0.244	0.057	0.080	AUC(-AAD-CDI)=0.805<br>AUC(-AAD+CDI)=0.941<br>AUC(-Ent-CDI)=0.818<br>AUC(-Ent+CDI)=0.965<br>AUC(+Ent-CDI)=0.945<br>AUC(+Ent+CDI)=0.912
**-AAD-CDI vs -AAD+CDI**	2	0.451	0.860	0.423	0.060	AUC(-AAD-CDI)=0.764<br>AUC(-AAD+CDI)=0.989
**-Ent-CDI vs -Ent+CDI**	2	0.506	0.487	0.236	0.005	AUC(-Ent-CDI)=0.656<br>AUC(-Ent+CDI)=0.947
**+Ent-CDI vs +Ent+CDI**	2	0.142	0.579	-0.210	1.000	AUC(+Ent-CDI)=0.730<br>AUC(+Ent+CDI)=0.947
**-Abx-CDI vs +Abx-CDI**	4	0.571	0.815	0.215	0.082	AUC(-AAD-CDI)=0.996<br>AUC(-Ent-CDI)=0.673
**-Abx+CDI vs +Abx+CDI**	2	0.431	0.856	0.339	0.594	AUC(-AAD+CDI)=0.982<br>AUC(-Ent+CDI)=0.850

a

b

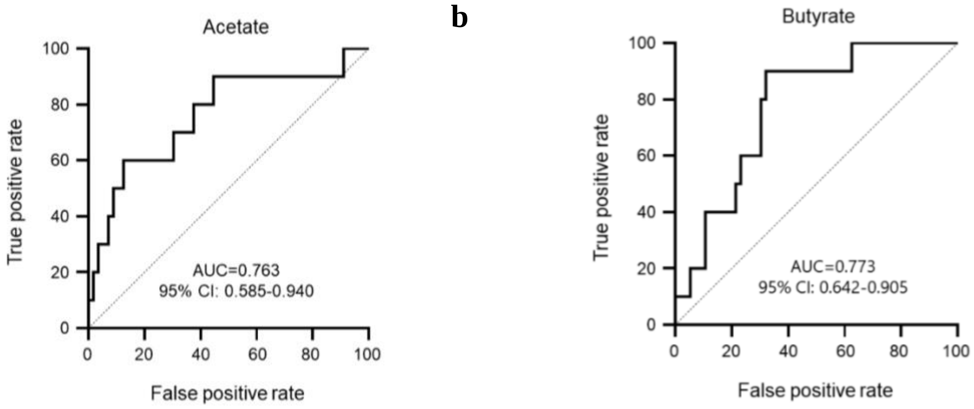

Supplementary Figure 3. Elevated acetate and butyrate were potential biomarkers of non-enterococcal CDI. a Acetate AUC plot differentiating +Ent-CDI (n=49) from +Ent+CDI (n=7) patients. **b** Butyrate AUC plot differentiating +Ent-CDI (n=49) from +Ent+CDI (n=7) patients.

Supplementary Table 4. Top ROC-AUC derived metabolite biomarkers (AUC ≥ 0.7) that differentiated the non-antibiotic associated CDI metabolome from non-antibiotic associated non-CDI metabolome.

Metabolite	AUC	p-value
**L-mimosine**	0.848	0.004
**Trans-3-hydroxy-L-proline**	0.829	0.019
**Glycolic acid**	0.819	0.017
**Cinnamic acid**	0.819	0.016
**3-phenyllactic acid**	0.819	0.028
**2-hydroxybutyric acid**	0.800	0.021
**Ribulose-5-phosphate**	0.800	0.031
**Hexanoic acid**	0.790	0.029
**L-proline**	0.790	0.027
**Ribose**	0.781	0.025
**Uric acid**	0.781	0.040
**2-hydroxyisovaleric acid**	0.781	0.015
**N-methylalanine**	0.771	0.031
**Pyruvic acid**	0.762	0.053
**Heptanoic acid**	0.762	0.037
**N-acetyl-D-mannosamine**	0.752	0.038
**Caffeic acid**	0.752	0.038
**L-tryptophan**	0.733	0.048

Supplementary Table 5. Top ROC-AUC derived metabolite biomarkers (AUC ≥ 0.7) that differentiated the antibiotic-associated CDI metabolome from the antibiotic-associated non-CDI metabolome.

Metabolite	AUC	p-value
**Beta-hydroxyisovalerate**	0.813	0.0012
**Xanthurenic acid**	0.800	0.0028
**Cholesterol**	0.792	0.0014
**Shikimic acid**	0.784	5.56×10^{-4}
**Xanthotoxin**	0.782	0.0116
**Adrenaline**	0.778	0.0165
**1,5-anhydro-D-sorbitol**	0.778	0.0091
**Glucoheptonic acid**	0.776	0.0066
**Conduritol epoxide**	0.761	0.0042
**Pantothenic acid**	0.761	0.0014
**Stearic acid**	0.761	0.0131
**Urocanic acid**	0.759	0.0016
**Arabinose**	0.752	0.0124
**3-chloro-L-tyrosine**	0.752	0.0067
**D-saccharic acid**	0.750	0.0300
**Galactonic acid**	0.746	0.0016
**Mucic acid**	0.746	0.0196
**L-asparagine**	0.741	0.0218
**L-sorbose**	0.740	0.0086
**D-glucose**	0.740	0.0312
**D(+) galactose**	0.738	0.0321
**Malonic acid**	0.736	0.0358
**Xanthine**	0.735	0.0065
**Gluconic acid**	0.731	0.0035
**5alpha-Cholestan-3-beta-ol**	0.730	0.0185
**Hippuric acid**	0.727	0.0375
**L-cystine**	0.725	0.0467
**Desaminotyrosine**	0.720	0.0075
**Taurine**	0.716	0.0373
**Galacturonic acid**	0.716	0.0036
**Methyl-beta-D-galactopyranoside**	0.714	0.0085
**Trans-13-octadecenoic acid**	0.712	0.0417
**Glycine**	0.710	0.0242

Metabolite	AUC	p-value
**DL-4-hydroxy-3-methoxymandelic acid**	0.708	0.0187
**N-acetyl-D-mannosamine**	0.708	0.0426
**Salicylic acid**	0.707	0.0414
**Palmitoleic acid**	0.706	0.0136
**Citrulline**	0.705	0.0244

Supplementary Table 6. Top ROC-AUC derived metabolite biomarkers (AUC ≥ 0.7) that differentiated the enterococcal-dominant CDI metabolome from the non-enterococcal dominant CDI metabolome.

Metabolite	AUC	p-value
**Maltotriose**	0.795	0.033
**Beta-sitosterol**	0.786	0.010
**Taxifolin**	0.778	0.007
**Inosine**	0.760	0.012
**Uracil**	0.728	0.098
**Citraconic acid**	0.728	0.098
**Maltose**	0.722	0.070
**N-methylalanine**	0.720	0.061
**Iminodiacetic acid**	0.717	0.075
**Methoxytryptamine**	0.717	0.081
**5-aminovaleric acid**	0.735	0.027
**Tannic acid**	0.714	0.043
**Thymine**	0.706	0.073

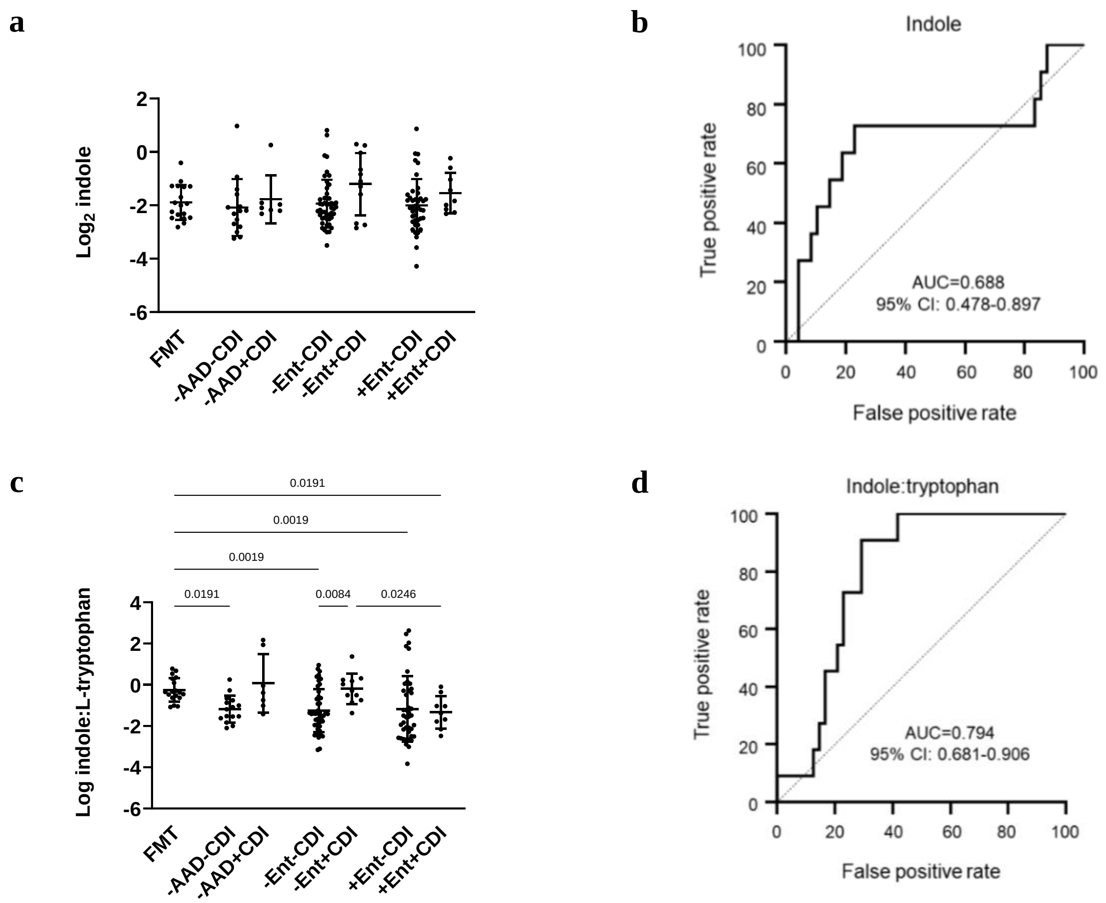

Supplementary Figure 4. Non-enterococcal CDI patients were associated with elevated indole/tryptophan ratios. a Dot plot of indole abundance for FMT donors (n=18), -AAD-CDI (n=15), -AAD+CDI (n=7), -Ent-CDI (n=48), -Ent+CDI (n=11), +Ent-CDI (n=42) and +Ent+CDI (n=9) patients. **b** Indole AUC plot differentiating +Ent-CDI (n=42) from +Ent+CDI (n=9) patients. **c** Dot plot of indole/L-tryptophan ratios. **d** Indole/tryptophan ratios AUC plot differentiating +Ent-CDI (n=42) from +Ent+CDI (n=9) patients. Data in panels **a** and **c** are presented as mean ± SD. Statistical significance was determined at $p < 0.05$ and comparisons used Kruskal-Wallis tests with FDR adjusted for multiple comparisons using the Benjamini and Hochberg method.

Ratios of indole to tryptophan in non-enterococcal CDI

By-products of L-tryptophan Stickland fermentation (indolepropionic and indole-3-acetic acids) were also detected in the current study but were not considered significant metabolites in the CDI PLS-DA model (Supplementary Table 7). However, indole, a bacterial tryptophan

metabolite, was significant in the CDI PLS-DA model alongside host-derived tryptophan metabolites associated with the kynurenine pathway (Supplementary Table 7).

Indole abundance decreased with antibiotic usage and enterococcal dominance compared to FMT donors (Supplementary Figure 4A). Only -Ent+CDI patients showed a mean indole abundance that exceeded FMT donors and their non-CDI counterparts, but the differences were not considered significant ($p>0.05$) (Supplementary Figure 4A). Furthermore, univariate AUC biomarker analysis also determined that with an AUC <0.70, indole was not a significant biomarker differentiating -Ent+CDI patients from -Ent-CDI patients (Supplementary Figure 4B).

To investigate whether reduced L-tryptophan and elevated indole might signify microbiota activity, the ratio of indole to L-tryptophan was calculated for each individual. Compared to FMT donors, indole/L-tryptophan ratios were significantly decreased in -AAD-CDI ($p=0.019$), -Ent-CDI ($p=0.002$), +Ent-CDI ($p=0.002$) and +Ent+CDI ($p=0.019$) patients but were similarly elevated in -AAD+CDI, and -Ent+CDI patients (Supplementary Figure 4C). Only the ratio differences between -Ent+CDI and -Ent+CDI patients were statistically significant ($p=0.008$). Univariate AUC biomarker analysis determined that, with an AUC=0.794, indole/tryptophan ratios performed better in differentiating -Ent+CDI patients from -Ent-CDI patients than indole on its own (Supplementary Figure 4D).

Supplementary Table 7: Significant metabolites derived from the CDI PLS-DA model [1]

Metabolite	p(corr)	VIP	FC -AAD-CDI vs -AAD+CDI	FC -Ent-CDI vs -Ent+CDI	FC +Ent-CDI vs +Ent+CDI
2-hydroxy-3-methylvaleric acid	0.621	1.427	0.561	0.943	1.469
2-hydroxybutyric acid	0.810	1.880	0.626	1.041	1.014
2-hydroxyisovaleric acid	0.734	1.998	0.503	0.935	1.269
3,4-dihydroxybenzoic acid	-0.573	1.651	1.164	0.900	1.024
3-chloro-L-tyrosine	-0.689	1.632	0.775	0.663	0.980
3-phenyllactic acid	0.721	1.438	0.577	0.713	1.089
4-methylvaleric acid	0.531	2.409	0.689	1.445	1.028
5-aminovaleric acid	0.606	2.285	0.665	1.879	16.298
5-hydroxyindole-3-acetic acid	-0.712	1.349	0.944	0.832	0.895
6-hydroxy caproic acid	0.736	1.266	0.700	0.797	0.725
8-aminocaprylic acid	0.728	2.918	0.931	1.677	1.403
Adipic acid	0.683	1.398	0.534	0.906	1.377
Arabinose	-0.802	1.552	0.711	0.534	1.692
Aspartic acid	0.828	1.483	0.331	0.333	12.296
Benzoin	-0.533	2.026	1.185	1.040	0.940
Beta-hydroxyisovalerate	-0.733	2.398	0.655	0.326	1.695
Cadaverine	0.684	1.861	1.227	1.347	1.483
Caffeic acid	0.829	1.523	0.547	0.782	1.169
Cholesterol	-0.685	2.326	1.109	0.636	1.037
Cinnamic acid	0.681	1.870	0.686	0.884	1.008
Citrulline	-0.826	1.549	0.669	0.542	1.520
D(+) galactose	-0.830	1.418	0.571	0.335	0.585
D-glucose	0.874	1.368	0.435	0.327	0.585
Desaminotyrosine	0.573	2.424	1.058	1.517	0.965
DL-3-aminoisobutyric acid	0.599	2.146	0.817	1.176	1.152
DL-4-hydroxy-3-methoxymandelic acid	-0.514	2.748	0.605	0.591	0.594
DL-threo-beta-hydroxyaspartic acid	0.727	1.420	0.668	0.681	1.109
D-mannitol	0.599	1.337	0.613	0.628	0.766
D-sorbitol	-0.640	1.585	0.579	0.646	0.700
D-threitol	0.650	1.366	0.544	0.703	1.663
Elaidic acid	-0.585	1.587	1.294	0.689	0.846
Ferulic acid	-0.819	1.367	0.773	0.615	0.783
Galactitol	0.578	1.671	0.532	0.506	0.773
Galactonic acid	-0.809	1.861	0.597	0.369	0.636
Galacturonic acid	0.581	1.594	0.340	0.353	0.962
Glucoheptonic acid	-0.826	1.685	0.737	0.651	0.827
Gluconic acid	-0.802	1.716	0.597	0.402	0.684
Glycine	-0.773	1.686	0.576	0.485	1.949
Glycolic acid	0.650	1.917	0.264	0.588	0.777
Heptadecanoic acid	-0.549	1.877	1.554	0.794	0.813
Heptanoic acid	0.687	1.704	0.628	0.838	1.088
Hexanoic acid	0.733	1.830	0.538	0.917	0.757

Metabolite	p(corr)	VIP	FC<br>-AAD-CDI<br>vs<br>-AAD+CDI	FC<br>-Ent-CDI<br>vs<br>-Ent+CDI	FC<br>+Ent-CDI<br>vs<br>+Ent+CDI
**Hippuric acid**	-0.684	1.548	0.919	0.709	0.949
**Hypoxanthine**	-0.849	1.612	0.593	0.497	1.460
**Indole**	0.616	2.663	1.176	1.606	1.300
**Inosine**	-0.541	3.330	0.699	0.765	0.639
**Itaconic acid**	-0.612	1.307	0.453	0.325	2.867
**L-sorbose**	-0.588	2.277	0.568	0.562	0.733
**L-(+) lactic acid**	0.775	1.444	0.370	0.603	0.237
**Lactitol**	-0.761	1.399	0.810	0.676	0.835
**Lactose**	-0.849	1.353	0.773	0.706	0.732
**Lactulose**	-0.518	2.223	1.008	0.760	0.528
**L-alanine**	0.576	1.433	0.176	0.505	12.056
**L-asparagine**	-0.805	1.417	0.651	0.559	1.231
**L-cystine**	-0.794	1.405	0.856	0.778	1.052
**L-leucine**	0.823	1.258	0.357	0.001	2.429
**L-methionine**	0.568	1.904	0.505	0.712	2.139
**L-mimosine**	0.538	2.832	0.463	0.674	0.743
**L-ornithine**	0.518	1.650	0.754	0.964	3.239
**L-proline**	0.781	1.491	0.117	0.026	1.930
**L-tryptophan**	0.773	1.697	0.491	0.683	3.986
**L-tyrosine**	0.913	1.432	0.138	0.174	0.949
**Malonic acid**	-0.721	1.695	0.556	0.532	1.467
**Maltotriitol**	-0.657	2.593	0.823	0.890	0.669
**Maltotriose**	-0.505	2.953	1.016	0.802	0.676
**Methoxytryptamine**	0.524	2.063	1.015	1.677	1.926
**Mucic acid**	-0.818	1.414	0.669	0.624	0.853
**N-acetyl-D-mannosamine**	0.872	1.539	0.531	0.637	0.852
**N-acetylneuraminic acid**	-0.509	1.621	0.863	1.051	0.708
**N-acetyl-ornithine**	0.691	1.913	1.063	1.343	1.469
**N-ethylglycine**	0.672	1.757	0.521	1.316	1.523
**N-methylalanine**	0.602	2.154	0.536	1.357	2.013
**Oleic acid**	-0.585	1.589	1.294	0.703	0.852
**Palatinitol**	0.525	1.602	0.708	0.755	0.996
**Palatinose**	0.552	1.316	0.749	0.778	0.970
**Palmitoleic acid**	-0.808	1.717	0.471	0.445	0.776
**Pantothenic acid**	-0.784	1.990	0.490	0.249	0.823
**Phenethylamine**	0.727	1.391	0.486	1.047	2.213
**Phenylpropanolamine**	0.574	1.433	0.692	0.963	0.902
**Picolinic acid**	-0.641	2.303	1.324	0.901	0.951
**Putrescine**	0.797	2.855	0.791	1.823	1.574
**Pyruvic acid**	0.848	1.609	0.312	0.804	0.976
**Quinic acid**	-0.715	1.622	0.717	0.623	0.647
**Ribose**	0.749	1.514	0.698	0.798	1.001
**Ribulose-5-phosphate**	0.926	1.450	0.451	0.512	0.625
**Salicylurate**	-0.590	1.370	0.924	0.832	1.009

Metabolite	p(corr)	VIP	FC<br>-AAD-CDI<br>vs<br>-AAD+CDI	FC<br>-Ent-CDI<br>vs<br>-Ent+CDI	FC<br>+Ent-CDI<br>vs<br>+Ent+CDI
**Serotonin**	0.691	1.913	1.063	1.343	1.449
**Shikimic acid**	-0.700	2.477	0.771	0.492	0.854
**Stearic acid**	-0.641	1.488	0.930	0.746	1.051
**Sucrose**	-0.668	1.532	0.856	0.726	0.727
**Tannic acid**	-0.545	1.541	0.833	0.748	0.778
**Trans-3-hydroxy-L-proline**	0.830	1.725	0.627	0.836	1.283
**Trans-4-hydroxy-L-proline**	0.798	1.480	0.674	0.987	1.674
**Uric acid**	0.914	1.465	0.446	0.572	0.561
**Urocanic acid**	-0.787	1.954	0.488	0.264	0.837
**Xanthine**	-0.815	1.577	0.532	0.402	0.722
**Xanthotoxin**	-0.700	1.440	0.917	0.720	0.992
**Xanthurenic acid**	-0.710	1.917	0.807	0.570	1.068

[1] All metabolites were normalised, Pareto scaled, and log-transformed. Each metabolite loading was scaled as a correlation co-efficient p(corr) between the metabolomics model and the original data. Metabolites with VIP scores > 1.0 and p(corr) values > 0.5 and < -0.5 were identified as a subset of metabolites with the highest potential as biomarkers.

Supplementary Table 8. Quality control (QC) mix metabolites (n = 16) processed through GC-MS for quality check as determined by the percent relative standard deviation.

**QC metabolite**	**% RSD**
^{15}N L-glutamine	3.73
Palmitic acid	5.42
Valine	2.49
Salicylic acid	5.37
L-Methionine	2.78
L-Hydroxyproline	2.64
Shikimic acid	3.05
Citric acid	3.24
Sucrose	3.26

Supplementary references

1 Girinathan, B. P. *et al. In vivo* commensal control of *Clostridioides difficile* virulence. *Cell Host Microbe* **29**, 1693-1708.e1697, doi:10.1016/j.chom.2021.09.007 (2021).

2 Fiehn, O. Metabolomics by Gas Chromatography-Mass Spectrometry: Combined Targeted and Untargeted Profiling. *Curr Protoc Mol Biol* **114**, 30.34.31-30.34.32, doi:10.1002/0471142727.mb3004s114 (2016).

3 Karpe, A. V., Beale, D. J., Morrison, P. D., Harding, I. H. & Palombo, E. A. Untargeted metabolic profiling of Vitis vinifera during fungal degradation. *FEMS Microbiol Lett* **362**, doi:10.1093/femsle/fnv060 (2015).

4 Karpe, A. V. *et al.* Cryptosporidiosis Modulates the Gut Microbiome and Metabolism in a Murine Infection Model. *Metabolites* **11**, doi:10.3390/metabo11060380 (2021).

5 Beale, D. J. *et al.* Untargeted metabolomics analysis of the upper respiratory tract of ferrets following influenza A virus infection and oseltamivir treatment. *Metabolomics* **15**, 33, doi:10.1007/s11306-019-1499-0 (2019).

6 Fiehn, O. & Kind, T. Metabolite profiling in blood plasma. *Methods Mol Biol* **358**, 3-17, doi:10.1007/978-1-59745-244-1_1 (2007).

7 Chong, J. & Xia, J. Using MetaboAnalyst 4.0 for Metabolomics Data Analysis, Interpretation, and Integration with Other Omics Data. *Methods Mol Biol* **2104**, 337-360, doi:10.1007/978-1-0716-0239-3_17 (2020).

8 Furuhashi, T., Sugitate, K., Nakai, T., Jikumaru, Y. & Ishihara, G. Rapid profiling method for mammalian feces short chain fatty acids by GC-MS. *Analytical Biochemistry* **543**, 51-54, doi:https://doi.org/10.1016/j.ab.2017.12.001 (2018).
