# Supplementary material for: Multi-omics analysis of hospital-acquired diarrhoeal patients reveals biomarkers of enterococcal proliferation and Clostridioides difficile infection
Source: Nat Commun. 2023 Nov 25;14:7737. doi: 10.1038/s41467-023-43671-8 (PMC10676382; doi:10.1038/s41467-023-43671-8)
Supplement: Supplementary file 3 — Description of Additional Supplementary Files [file 41467_2023_43671_MOESM3_ESM.pdf]

Description of Additional Supplementary Files

Supplementary Data 1 : This dataset represents the metabolites detected via untargeted GC-MS and the raw peak areas for each metabolite for hospital-acquired diarrhoeal (HAD) and faecal microbiota transplant (FMT) donor samples.
