# Supplementary material for: Multi-omics analysis of hospital-acquired diarrhoeal patients reveals biomarkers of enterococcal proliferation and Clostridioides difficile infection
Source: Nat Commun. 2023 Nov 25;14:7737. doi: 10.1038/s41467-023-43671-8 (PMC10676382; doi:10.1038/s41467-023-43671-8)
Supplement: Supplementary file 5 — Reporting Summary [file 41467_2023_43671_MOESM5_ESM.pdf]

Corresponding author(s): Dena Lyras

Last updated by author(s): Nov 10, 2023

Reporting Summary

Nature Portfolio wishes to improve the reproducibility of the work that we publish. This form provides structure for consistency and transparency in reporting. For further information on Nature Portfolio policies, see our Editorial Policies and the Editorial Policy Checklist.

Statistics

For all statistical analyses, confirm that the following items are present in the figure legend, table legend, main text, or Methods section.

n/a	Confirmed	
☐	☒	The exact sample size (*n*) for each experimental group/condition, given as a discrete number and unit of measurement
☐	☒	A statement on whether measurements were taken from distinct samples or whether the same sample was measured repeatedly
☐	☒	The statistical test(s) used AND whether they are one- or two-sided<br>*Only common tests should be described solely by name; describe more complex techniques in the Methods section.*
☐	☒	A description of all covariates tested
☐	☒	A description of any assumptions or corrections, such as tests of normality and adjustment for multiple comparisons
☐	☒	A full description of the statistical parameters including central tendency (e.g. means) or other basic estimates (e.g. regression coefficient) AND variation (e.g. standard deviation) or associated estimates of uncertainty (e.g. confidence intervals)
☐	☒	For null hypothesis testing, the test statistic (e.g. *F*, *t*, *r*) with confidence intervals, effect sizes, degrees of freedom and *P* value noted<br>*Give P values as exact values whenever suitable.*
☒	☐	For Bayesian analysis, information on the choice of priors and Markov chain Monte Carlo settings
☒	☐	For hierarchical and complex designs, identification of the appropriate level for tests and full reporting of outcomes
☒	☐	Estimates of effect sizes (e.g. Cohen's *d*, Pearson's *r*), indicating how they were calculated

Our web collection on statistics for biologists contains articles on many of the points above.

Software and code

Policy information about availability of computer code

Data collection	DiaSorin LIAISON XL analyser (LIAISON XL,D iaSorin,S allugia,I taly)<br>Cepheid GeneXpert PCR platform (Cepheid International,S unnyvale,C alifornia,U SA)<br>NanoDrop 2000 Spectrophotometer (Thermo Fisher Scientific,W altham,M assachusetts,U SA)<br>lllumina MiSeq Sequencer (lllumina Inc,S an Diego,C alifornia,U SA)<br>QIIME v.l.9.1 (http:// qiime.org/index-qiimel.html)<br>Agilent 7890B gas chromatography system with 5977B mass spectrometry detector (Santa Clara,C alifornia,U nited States)<br>fitted with an MPS autosampler (Gerstel GmbH & Co. KG,M ulheim an der Ruhr,G ermany).<br>Shimadzu single quadrupole gas chromatograph-mass spectrometer (GCMS-QP2010,S himadzu,K yoto,J apan)<br>SPAdes genome assembler (https://github.com/ablab/spades)<br>Prokka (https:// github.com/tseemann/prokka)<br>MALDI-TOF MS (Bruker Corporation,B illerica,M assachusetts,U nited States)
Data analysis	IBM SPSS Statistics for Windows,v ersion 25 (IBM Corp.,A rmonk,N ew York,U SA)<br>Microsoft Excel for Microsoft 365 MSO (Version 2208 Build 16.0.15601.20660)<br>QIIME v.l.9.1 (http://qiime.org/index-qiimel.html)<br>Calypso software (http://cgenome.net/wiki/index.php/Calypso)<br>Graph Pad Prism version 8.2.1 for Windows (GraphPad Software,S an Diego,C alifornia,U SA)<br>Quantitative Analysis software (Version B.07.00) of the MassHunter workstation (Agilent Technologies,S anta Clara,U SA)<br>MetaboAnalyst 4.0 (https://www.metaboanalyst.ca)<br>SIMCA 16 Version 16 (Sartorius Stedim Biotech,U mea,S weden)<br>ClustVis (https:// biit.cs.ut.ee/clustvis)

I
GC-MS Post-run Analysis software (Shimadzu, Kyoto, Japan)
Nullabor v2.0 pipeline (https://github.com/tseemann/nullarbor)
Interactive Tree Of Life (iTOL) (https://itol.embl.de)

For manuscripts utilizing custom algorithms or software that are central to the research but not yet described in published literature, software must be made available to editors and reviewers. We strongly encourage code deposition in a community repository (e.g. GitHub). See the Nature Portfolio guidelines for submitting code & software for further information.

Data

Policy information about availability of data

All manuscripts must include a data availability statement. This statement should provide the following information, where applicable:

- Accession codes, unique identifiers, or web links for publicly available datasets
- A description of any restrictions on data availability
- For clinical datasets or third party data, please ensure that the statement adheres to our policy

The sequence data used for analysis is available in NCBI under BioProject accession number PRJNA986597 and PRJNA1015000. Sequence data is available.
Data is publically available. The raw 16S rRNA sequence data generated in this study have been deposited in the NCBI database under BioProject PRJNA986597, accession numbers SRR249990219- SRR24999022.
https://www.ncbi.nlm.nih.gov/sra/?term=SRR24999019, https://www.ncbi.nlm.nih.gov/sra/?term=SRR24999020; https://www.ncbi.nlm.nih.gov/sra/?term=SRR24999021; https://www.ncbi.nlm.nih.gov/sra/?term=SRR24999022
The raw E. faecium sequence data generated in this study have been deposited in the NCBI database under
BioProject ID PRJNA1015000, accession numbers SAMN37345311- SAMN37345366.
https://www.ncbi.nlm.nih.gov/biosample/37345311; https://www.ncbi.nlm.nih.gov/biosample/37345312 https://www.ncbi.nlm.nih.gov/biosample/37345313; https://www.ncbi.nlm.nih.gov/biosample/37345314 https://www.ncbi.nlm.nih.gov/biosample/37345315; https://www.ncbi.nlm.nih.gov/biosample/37345316 https://www.ncbi.nlm.nih.gov/biosample/37345317; https://www.ncbi.nlm.nih.gov/biosample/37345318 https://www.ncbi.nlm.nih.gov/biosample/37345319; https://www.ncbi.nlm.nih.gov/biosample/37345320 https://www.ncbi.nlm.nih.gov/biosample/37345321; https://www.ncbi.nlm.nih.gov/biosample/37345322 https://www.ncbi.nlm.nih.gov/biosample/37345323; https://www.ncbi.nlm.nih.gov/biosample/37345324 https://www.ncbi.nlm.nih.gov/biosample/37345325; https://www.ncbi.nlm.nih.gov/biosample/37345326 https://www.ncbi.nlm.nih.gov/biosample/37345327; https://www.ncbi.nlm.nih.gov/biosample/37345328 https://www.ncbi.nlm.nih.gov/biosample/37345329; https://www.ncbi.nlm.nih.gov/biosample/37345330 https://www.ncbi.nlm.nih.gov/biosample/37345331; https://www.ncbi.nlm.nih.gov/biosample/37345332 https://www.ncbi.nlm.nih.gov/biosample/37345333; https://www.ncbi.nlm.nih.gov/biosample/37345334 https://www.ncbi.nlm.nih.gov/biosample/37345335; https://www.ncbi.nlm.nih.gov/biosample/37345336 https://www.ncbi.nlm.nih.gov/biosample/37345337; https://www.ncbi.nlm.nih.gov/biosample/37345338
The metabolomics data generated in this study are provided in the Supplementary Data file and Source Data file.

Research involving human participants, their data, or biological material

Policy information about studies with human participants or human data. See also policy information about sex, gender (identity/presentation), and sexual orientation and race, ethnicity and racism.

Reporting on sex and gender	The data on sex was collected from hospital admission records as part of our clinical sample collection and reported on in our paper under demographic data. However sex was not used as as a factor in our analysis.
Reporting on race, ethnicity, or other socially relevant groupings	The study did not report on race, ethnicity, or other socially relevant groupings
Population characteristics	Population characteristics collected in the study included: age, comorbidities, unit of admission, antibiotic treatment prior to sample collection and period of antibiotic usage prior to sample collection.
Recruitment	As a retrospective sample collection for a microbiology lab, recruitment was based on the assessment of whether a) is it diarrhoea b) if yes, did it develop 3 days post hospitilisation, and c) if yes, is the patient over 18. yes, then eligible. See supplementary materials. All diarrhoeal samples were selected from hospitalised patients who developed diarrhoeal after two or more days of hospitalisation. Samples were selected retrospectively at random from May 2017-March 2018 following laboratory analysis. Only those samples that fulfilled the inclusion criteria we retained. Faecal Microbiota Tranplant patient samples were selected at random by our collaborators at the BiomeBank.<br>While sample collection was undertaken over a period of 12 months, biases relate to interpretation of Bristol Stool Chart.
Ethics oversight	Approval for the use of FMT specimens was obtained from Bellberry Human Research Ethics Committee (HREC 2020-03-288). Monash University Human Research Ethics Committee (HREC 29548) also approved the use of these specimens. Approval for the use of HAD specimens was obtained from Monash Health Human Research Ethics Committee (HREC 49004) and Monash University Human Research Ethics Committee (HREC 28455). Donor, patient and specimen evaluations for these cohorts are found in Supplementary Materials. All participants consented to this study.

Note that full information on the approval of the study protocol must also be provided in the manuscript.

Field-specific reporting

Please select the one below that is the best fit for your research. If you are not sure, read the appropriate sections before making your selection.

☒ Life sciences ☐ Behavioural & social sciences ☐ Ecological, evolutionary & environmental sciences

For a reference copy of the document with all sections, see nature.com/documents/nr-reporting-summary-flat.pdf

Life sciences study design

All studies must disclose on these points even when the disclosure is negative.

Sample size

169 diarrhoeal samples and 20 faecal microbiota transplant samples were collected for this study. a point prevalence study of 485 hospitalised patients found that up to 12% of patients had diarrhoea (Polage et al, 2012). Based on these proportions, we calculated that a sample size of 169 was associated with a 95% CI (0.0803, 0.1597), SE of 0.0203 and RSE of 16.88 and was deemed sufficient for this study.

Data exclusions

Hospital diarrhoeal sample exclusion criteria were as follows:

All donor samples in the study were from individuals between the ages of 18 and 98 who had no record of:
• A specific pathogen (other than C. difficile) was identified from the diarrhoea faecal sample during routine diagnostic testing
Exclusions included:
• Formed stool
• Sample submitted was from outpatient or emergency departments
• Sample submitted< 48 hours after admission
• Repeat specimen (only first sample from patient's index admission processed)

Faecal Microbiota Transplant sample exclusion criteria were as follows:
All donor samples in the study were from individuals between the ages of 18 and 65 and had no history of:
• Antimicrobial therapy or probiotics in the three months before donation
• Active medical illness or symptoms
• Medications (other than an oral contraceptive pill)
• International travel in the last six months
• High-risk sexual activity
• Illicit drug use
• Family history of colorectal carcinoma involving one or more first-degree relatives <55 years of age
• Household members with an active gastrointestinal infection
Medical exclusions included:
• Any gastrointestinal disorder
• Obesity (BMI >30) and malnutrition (BMI <18)
• Hypertension
• Type 1 and 2 diabetes
• Autoimmune disease
• Depression
• Malignancy
• Stroke
• Heart disease

Replication

This was an observational study over a set period of time that did not include replication. The sample size was significant in order to be able to characterise the microbiota and metabolome of hospital acquired diarrhoeal patients.

Randomization

Samples were selected retrospectively at random from May 2017-March 2018 following laboratory analysis. Only those samples that fulfilled the inclusion criteria were retained. Following selection based on diarrhoeal sample, HAD and age over 18, samples were then allocated into groups based on hospital patient data collection. To control the co-variates, we stratified the data with sub-group based factors such as antibiotic treatment, CDI and enterococcal co-infection.

Blinding

Blinding was not possible in this study. One investigator determined eligibility based on assessment of diarrhoea samples submitted to microbiology lab and a second investigator determined allocation into groups based on clinical data. All data was deidentified.

Reporting for specific materials, systems and methods

We require information from authors about some types of materials, experimental systems and methods used in many studies. Here, indicate whether each material, system or method listed is relevant to your study. If you are not sure if a list item applies to your research, read the appropriate section before selecting a response.

Materials & experimental systems

n/a	Involved in the study
☒	☐ Antibodies
☒	☐ Eukaryotic cell lines
☒	☐ Palaeontology and archaeology
☒	☐ Animals and other organisms
☐	☒ Clinical data
☒	☐ Dual use research of concern
☒	☐ Plants

Methods

n/a	Involved in the study
☒	☐ ChIP-seq
☒	☐ Flow cytometry
☒	☐ MRI-based neuroimaging

Clinical data

Policy information about clinical studies

All manuscripts should comply with the ICMJE guidelines for publication of clinical research and a completed CONSORT checklist must be included with all submissions.

Clinical trial registration	Faecal Microbiota Transplant samples - Bellberry Human Research Ethics Committee (HREC 2020-03-288) and Hospital Acquired Diarrheal Samples Monash Health Human Research Ethics Committee (HREC 49004) and Monash University Human Research Ethics Committee (HREC 28455).
Study protocol	The full study protocol is available in the Supplementary Materials.
Data collection	Hospital diarrhoeal samples were collected by our collaborators from Monash Health, Clayton, Victoria, Australia across all hospital units excluding the emergency department. Faecal Microbiota Transplant samples were collected in at the BiomeBank in Thebarton, Adelaide, Australia from volunteers in the Adelaide region.
Outcomes	We pre-defined the primary outcome measures as the proportions of microbial taxa in relation to C. difficile and antibiotic treatment and the secondary outcome measures as the differences in metabolite abundances that may or may not have been associated with these microbiota differences. These outcomes were measured by 16s RNA amplicon sequencing and gas-chromatography mass spectroscopy techniques.

Plants

Seed stocks	N/A
Novel plant genotypes	N/A
Authentication	N/A
